# Xenotransplantation of adult hippocampal neural progenitors into the developing zebrafish for assessment of stem cell plasticity

**DOI:** 10.1371/journal.pone.0198025

**Published:** 2018-05-24

**Authors:** Elizabeth J. Sandquist, Jeffrey J. Essner, Donald S. Sakaguchi

**Affiliations:** 1 Genetics, Development and Cell Biology, Iowa State University, Ames, Iowa, United States of America; 2 Neuroscience Program, Iowa State University, Ames, Iowa, United States of America; University of Colorado Boulder, UNITED STATES

## Abstract

Adult stem cells are considered multipotent, restricted to differentiate into a few tissue-specific cell types. With the advent of technologies which can dedifferentiate and transdifferentiate cell types, assumptions about the process of cell fate determination must be reconsidered, including the role of extrinsic versus intrinsic factors. To determine the plasticity of adult neural progenitors, rat hippocampal progenitor cells were xenotransplanted into embryonic zebrafish. These animals allow for easy detection of transplanted cells due to their external development and transparency at early stages. Adult neural progenitors were observed throughout the zebrafish for the duration of the experiment (at least five days post-transplantation). While the majority of transplanted cells were observed in the central nervous system, a large percentage of cells were located in superficial tissues. However, approximately one-third of these cells retained neural morphology and expression of the neuronal marker, Class III β-tubulin, indicating that the transplanted adult neural progenitors did not adapt alternate fates. A very small subset of cells demonstrated unique, non-neural flattened morphology, suggesting that adult neural progenitors may exhibit plasticity in this model, though at a very low rate. These findings demonstrate that the developing zebrafish may be an efficient model to explore plasticity of a variety of adult stem cell types and the role of external factors on cell fate.

## Introduction

Adult neural progenitors can differentiate into neurons, astrocytes and oligodendrocytes [1, 2]. However, it is unclear whether these multipotent cells can demonstrate expanded potential, or plasticity, under the proper circumstances. Further, the relative influence of intrinsic versus extrinsic factors restricting neural progenitor cell fate are relatively unknown. The survival and differentiation of multipotent progenitors after transplantation to the developing embryo provide insight on the external factors determining cell fate, which has implications for the therapeutic applications of stem cell research.

Previous research has indicated that adult neural progenitors can give rise to cells of ectodermal, mesodermal, and endodermal layers when transplanted into chick and mouse embryos, though these findings are dependent upon the origin of neural progenitors, such as brain region and donor age [3–6]. While transplantation and observation of cells transplanted in living mammalian and avian embryos is difficult and time-intensive, zebrafish embryos develop rapidly external to the mother and are transparent at early stages, providing the ability to track cells in a living organism over multiple time points. The zebrafish embryo is an ideal model system for the study of development, cell fate and plasticity.

Here, adult mammalian neural progenitors were xenotransplanted into embryonic zebrafish for the investigation of stem cell plasticity. Adult rat hippocampal progenitor cells expressing green fluorescent protein (AHPCs) were successfully transplanted into zebrafish embryos at the blastula stage and observed at least five days following. While transplanted cells were initially observed in multiple regions, the majority were located in the central nervous system (CNS) by 5 days post fertilization. A large proportion were also located at superficial regions of the larva such as epidermis and periderm, though most retained neural fates as determined by immunohistochemistry. A very low percentage of transplanted cells were observed with epithelial-like morphology.

## Methods and materials

### Zebrafish husbandry

An aquatic habitat system from Aquatic Ecosystems, Inc. was used to rear zebrafish. Zebrafish were maintained at 27°C with a 14 hr light/ 10 hr dark cycle. Zebrafish strains used included Casper [7], Tg(flk:mCherry-β-actin) [8] and Wik (Zebrafish International Resource Center, Eugene OR). Embryos were incubated at 28.5°C in fish water (60.5 mg ocean salts/L). Zebrafish embryos were staged according to published guidelines [9]. Fish were anesthetized with 200 mg/L tricaine methanesulfonate (Syndel USA, Western Chemical, Ferndale, WA) and euthanized by tricaine overdose.

Animals were reared and euthanized in accordance with protocol # 11-06-6252-I approved by the Iowa State University Institutional Animal Care and Use Committee. All protocols were in compliance with the American Veterinary Medical Association and the National Institutes of Health guidelines for the humane use of laboratory animals in research.

### Cell culture

Adult rat hippocampal progenitor cells (AHPCs) were isolated as previously described [1] and retrovirally infected to express green fluorescent protein (GFP) [10], a gift from F.H. Gage (Salk Institute for Biological Sciences, La Jolla, CA). Cells were maintained in flasks coated with poly-L-ornithine (10 μg/ml, Sigma-Aldrich, St. Louis, MO) and purified mouse laminin (5 μg/mL Biotechne, Minneapolis, MN) in Earle’s Balanced Salt Solution (EBSS, Thermo Fisher Scientific, GIBCO, Waltham, MA). Cells were cultured in maintenance media containing Dulbecco’s Modified Eagle’s Medium (Corning, Mediatech, Corning, NY) and Ham’s F-12 (1:1, Omega Scientific, Tarzana, CA) supplemented with 2.5 mM L-glutamine, 1x N2 (Thermo Fisher Scientific, GIBCO, Waltham, NY) and 20 ng/ml basic fibroblast growth factor (human recombinant bFGF, Promega Corp, Madison, WI). Half the volume of maintenance media was replaced every other day, and cells were maintained at 37^o^ C in 5% CO_2_/95% humidified air. Cells were harvested for transplant by detachment with 0.05% trypsin-EDTA (Thermo Fisher Scientific, Waltham, MA) followed by centrifugation at 800 rpm for 5 min and resuspension in EBSS.

### Cell transplantation

Embryos were enzymatically dechorionated at 3 hours post fertilization (hpf) by incubation for seven minutes in 0.2% trypsin (Sigma-Aldrich, St. Louis, MO) followed by one wash in 5% sheep serum and several rinses with fish water. Embryos were then transferred to an agarose (Thermo Fisher Scientific, Waltham, MA) injection tray containing wedge-shaped troughs made with a plastic mold [11]. The tray was filled with 0.5% penicillin-streptomycin (Gibco, Waltham, MA) in fish water for transplantation [11]. Glass micropipettes were pulled using a Flaming-Brown pipette puller and beveled using a K.T. Brown Type micro-pipette beveler (Sutter Instrument, Novato, CA). Adult hippocampal progenitor cells suspended in EBSS were transplanted to embryos between 3 and 4 hpf from the animal pole to the center of the blastoderm with guidance of a Narashigi micromanipulator and dissecting scope. Approximately 20 to 100 cells were transplanted to each embryo. Embryos were allowed to recuperate for approximately 20 minutes, after which they were transferred to agarose-coated trays containing 0.5x penicillin-streptomycin in fish water and grown at 34°C.

### Whole-mount immunohistochemistry

Embryos and larvae were sacrificed at 1, 3 and 5 days post fertilization (dpf) by tricaine overdose and fixed with 4% paraformaldehyde (Sigma-Aldrich, St. Louis, MO) in 0.1 M phosphate buffer (1 dpf embryos) or 3% trichloroacetic acid (3 and 5 dpf) (Thermo Fisher Scientific, Waltham, WA) for three hours at room temperature (RT). Embryos and larvae were then dehydrated in a series of washes using 25% ethanol in phosphate-buffered saline (PBS), 50% ethanol in PBS, 75% ethanol in ddH20, and 100% ethanol. Samples were stored at -20^o^ C. Embryos and larvae were then rehydrated prior to immunohistochemical procedures using the ethanol series in reverse. Samples were then washed in 0.1% Triton X-100 Thermo Fisher Scientific, Waltham, WA) in PBS (PBS-T) and 3 and 5 dpf larvae were incubated in fresh 0.25% trypsin in PBS for 9 minutes on ice. Samples were then blocked in a solution of 1% dimethyl sulfoxide (DMSO) (Thermo Fisher Scientific, Waltham, WA), 1% BSA (Sigma-Aldrich, St. Louis, MO), and 5% normal donkey serum (Jackson ImmunoResearch, West Grove, PA) in PBS-T for one hour. Embryos and larvae were incubated in primary antibodies at 1:50 for three days at 4^o^ on a nutator. The following primary antibodies were used: polyclonal rabbit anti-GFP (sc-8334, Santa Cruz Biotechnology, Dallas, TX), monoclonal mouse anti-rat 401 for nestin (Developmental Studies Hybridoma Bank (DSHB) Iowa City, IA), monoclonal mouse anti-Class III β-tubulin (TuJ1) (MAB 1195, Biotechne, R&D Systems, Minneapolis, MN), monoclonal mouse anti-glial fibrillary acidic protein (MAB360, EMD Millipore, Billerica, MA), and monoclonal mouse anti-RIP (DSHB, Iowa City, IA). Samples were then washed eight times for 15 minutes each in 1% DMSO, 1% BSA in PBS-T and incubated overnight in a cocktail of secondary antibody (1:500) and DAPI (1:50 Life Technologies, Carlsbad, CA). The secondary antibodies used were donkey anti-mouse Cy3 and donkey anti-rabbit AF488 (Jackson ImmunoResearch, West Grove, PA). Embryos and larvae were washed 8 times for 15 min with PBS and transferred to 70% glycerol (Thermo Fisher Scientific, Waltham, WA) in PBS for imaging.

### Confocal microscopy and image analysis

Embryos and larvae were visualized using a Zeiss LSM 700 Imager Z2. Z-stacks were captured at 10 and 20x. The left and right sides of each sample were analyzed as z-stacks and maximum intensity projections were used to determine cell localization and immunolabeling using ImageJ software version 1.47v (NIH, Bethesda, MD; http://imagej.nih.gov/ij). Cell location was quantified as the average percent of cells per location per fish. Statistical analysis was performed using GraphPad Prism.

## Results

While zebrafish grow optimally at 29°C, mammalian cells are cultured at a warmer temperature of 37°C. Therefore, zebrafish were maintained at an intermediate temperature of 34°C following transplantation. Experiments comparing zebrafish grown at 29 or 34°C showed little to no difference, as indicated by survival and average fish length (S1 Fig, S1 and S2 Tables).

**Fig 1:**
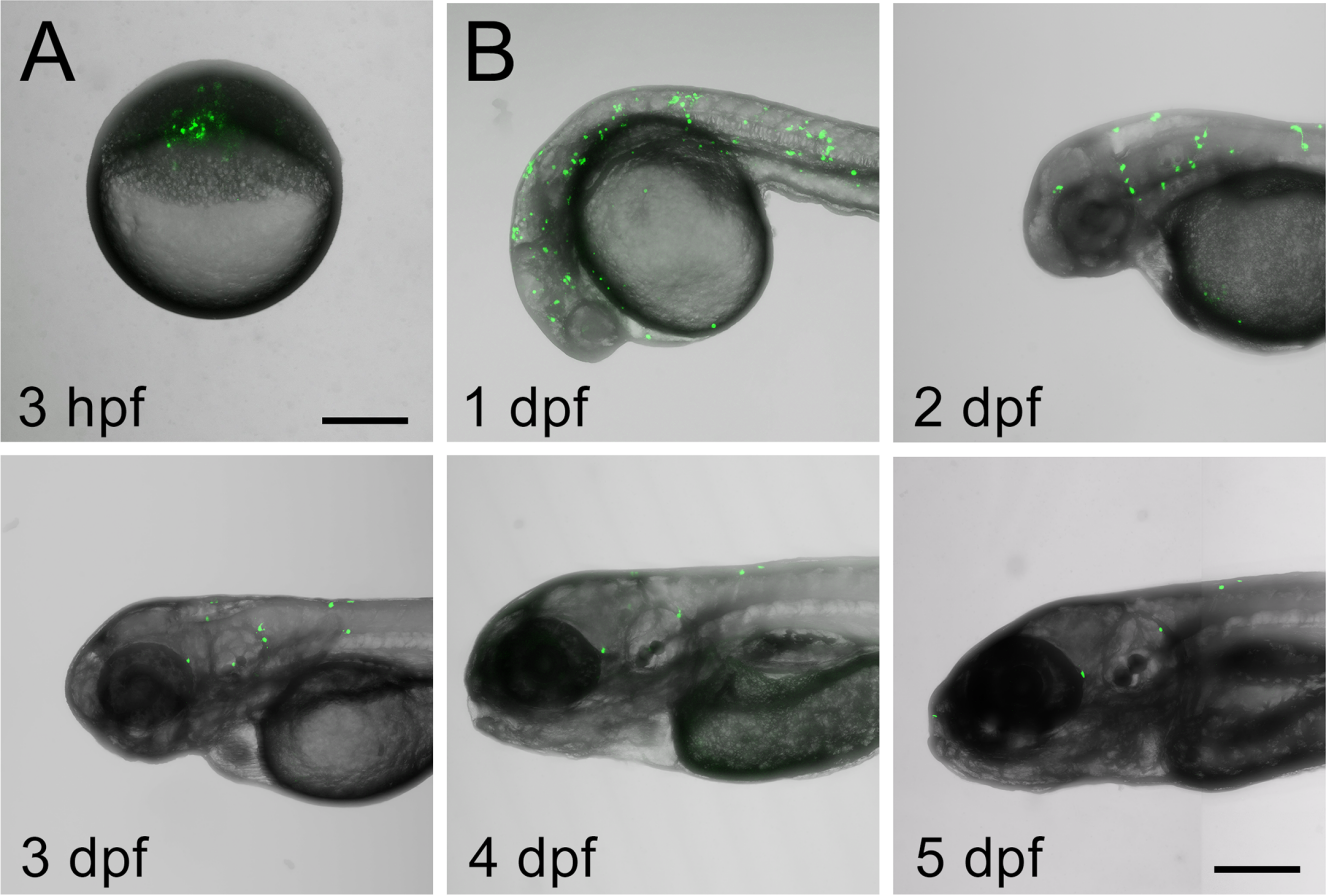
Adult hippocampal progenitor cells transplanted at blastula stage are observed at least 5 days post-transplantation. A) Representative image of blastula containing transplanted cells. B) Zebrafish with transplanted cells at 1, 2, 3, 4 and 5 dpf. Green = GFP-expressing AHPCs. Scale bar = 250 μm.

Transplantation of AHPCs was performed at blastula stage by injection from the animal side of the blastoderm. Homogeneity of the cell population was characterized by positive labeling for the neural progenitor marker nestin in 86% of cells (S3 Fig and S3 Table). Embryos were then maintained at 34°C for up to five days. Mortality was highest in the first 24 hours following transplantation with a survival rate of 71% (N = 164), but the majority of remaining embryos with AHPCs survived at least five days post-transplantation (Fig 1 and S2 Fig). Of 47 embryos observed at multiple time points up to 5 dpf, 69% still contained GFP-positive cells at 5 dpf. The number of transplanted cells per fish was not significantly different between 1 dpf (Mean = 24.5, SD = 30.96, N = 8), 3 dpf (Mean = 14.46, SD = 17.52, N = 13) and 5 dpf (Mean = 5.67, SD = 4.9, N = 9) due to high variability, though loss of cells was observed between 3 and 5 dpf.

**Fig 2:**
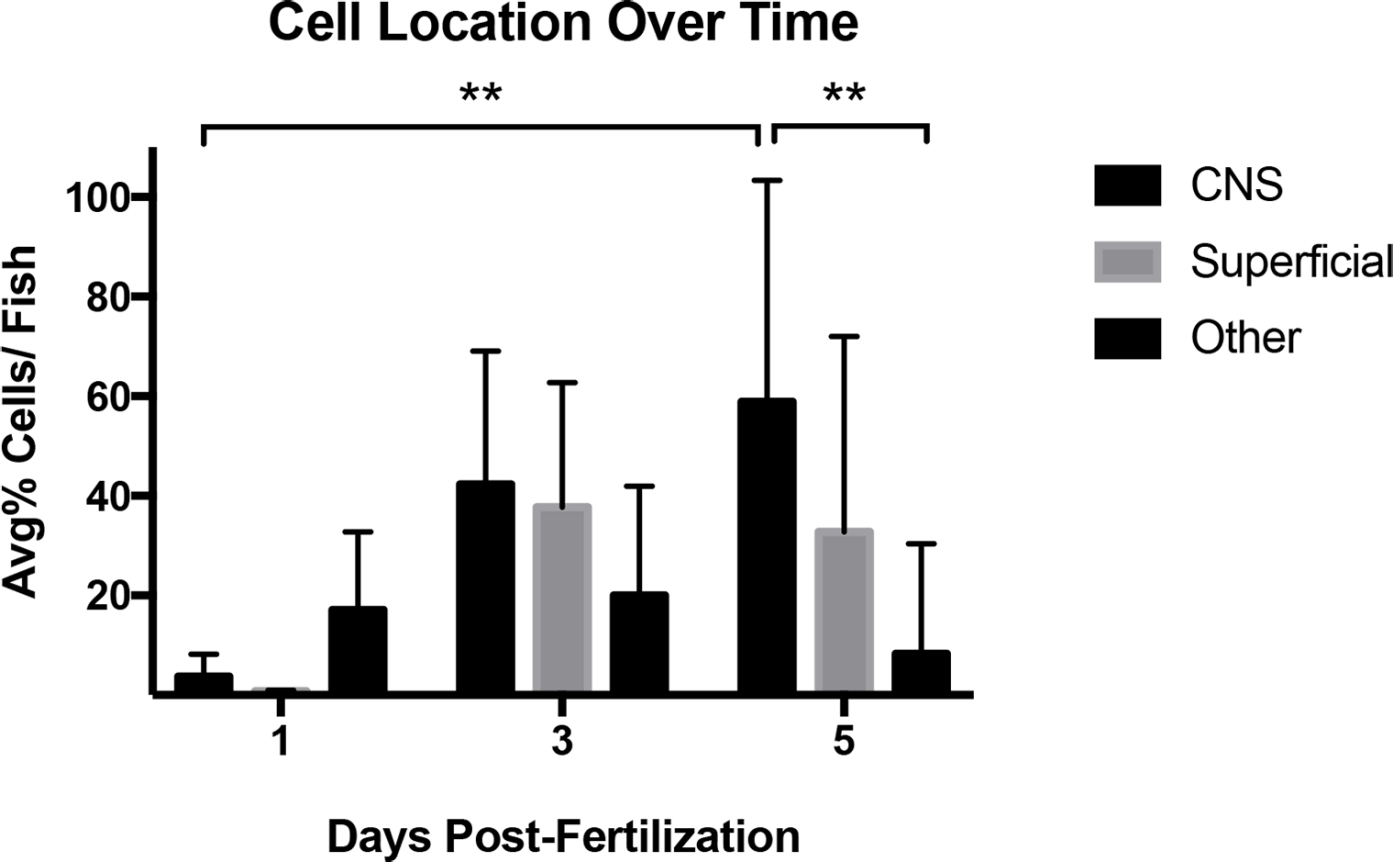
Transplanted cells are retained in the CNS and superficial regions over time. Data represents the average percent of transplanted cells per fish at each location over time. At 5 dpf, a greater percentage of transplanted AHPCS were found in the CNS than in other non-superficial regions, and a greater proportion of cells were found in the CNS than at 1 dpf. **p≤ 0.01 Two-way ANOVA with Tukey’s multiple comparisons test. N = 6–13 animals per time point. Error bars represent standard error of the mean.

The location of AHPCs in each fish was quantified at 1, 3 and 5 dpf using whole mount confocal images of fixed animals. Cells were categorized as CNS (brain, neural tube and retina), superficial, (within or just under the epidermis and the yolk periderm) or other (muscle, gut, or cartilage). At 1 and 3 dpf, the average percent of cells per fish at each location was not statistically different due to high variability. However, at 5 dpf, a significantly greater percent of cells per fish were observed in the CNS (p≤ 0.01, Mean = 58.9, SD = 44.5, N = 9) compared to the other category (Mean = 8.3, SD = 22, N = 9). A large proportion of cells were also observed at superficial locations (Mean = 32.8, SD = 39.2, N = 9) (Fig 2 and S4 Table).

**Fig 3:**
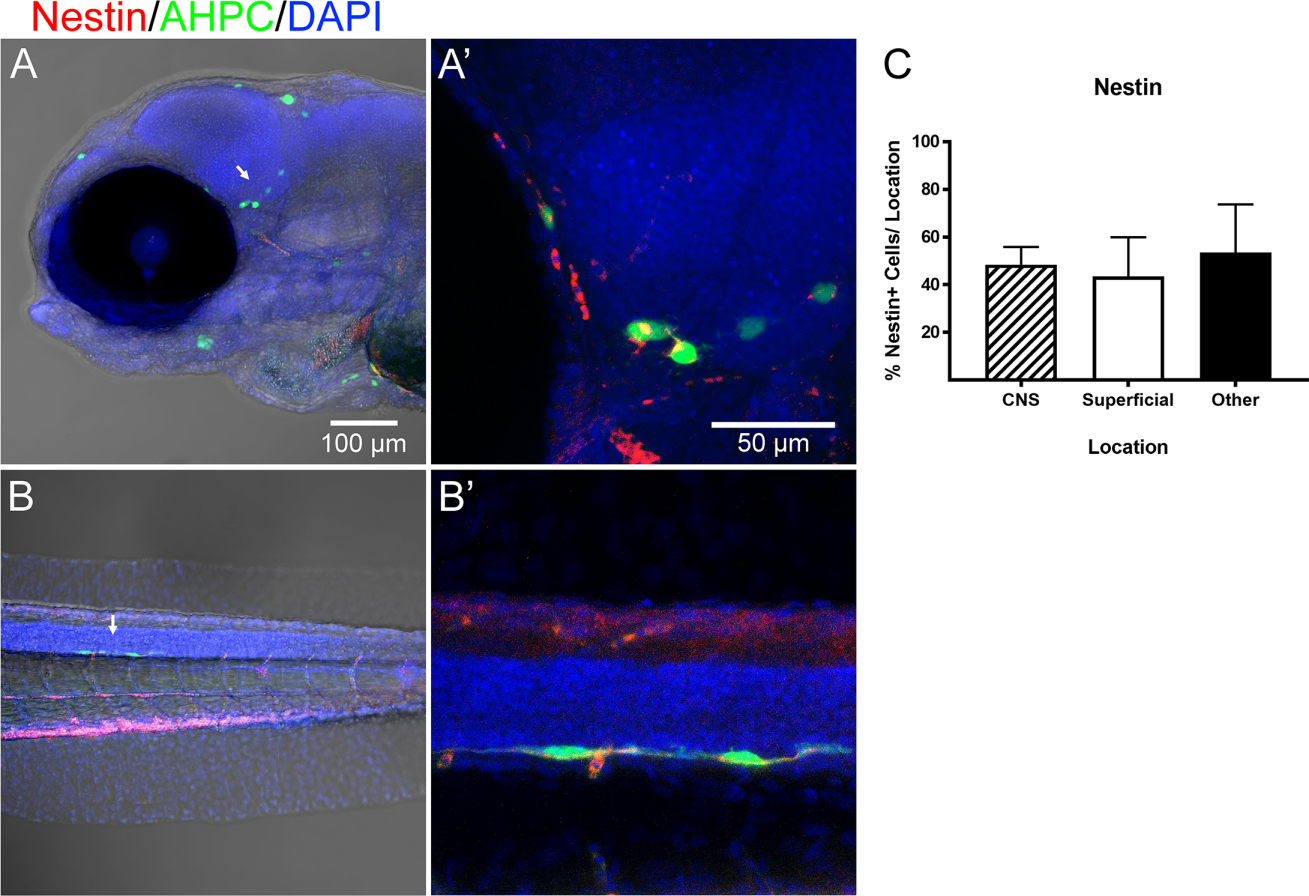
A large percentage of transplanted cells retain neural progenitor phenotypes. Larvae at 3 dpf with transplanted AHPCs were immunolabeled for Nestin (red) at 3 dpf. Arrows indicate cells selected for higher magnification. A) Cells located at CNS and superficial regions were positive for Nestin. B) Cells in the zebrafish tail were Nestin positive. C) Quantification of average percent of Nestin^+^ cells/ location per fish at 3 dpf. N = 6. Error bars represent standard error of the mean.

**Fig 4:**
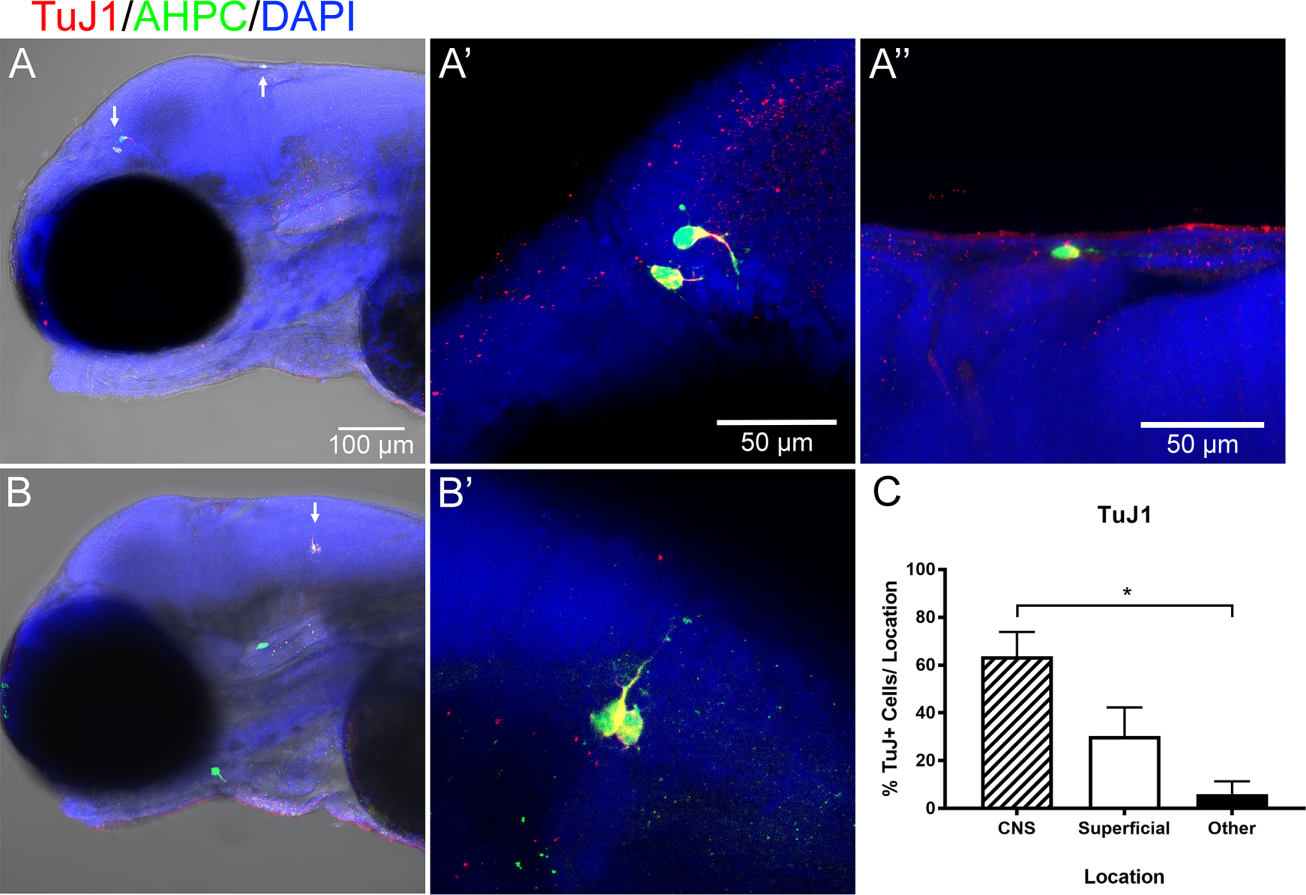
Transplanted cells in the CNS adopted a neuronal fate. A significant proportion of superficially-located cells were also neuronal, as indicated by TuJ1 immunolabeling (red) at 3 dpf. Arrows indicate cells selected for higher magnification. A) TuJ1^+^ cells were in the brain and at a superficial region. B) TuJ1^+^ cells in the brain and TuJ1^-^ cells in facial cartilage. C) Quantification of the percent of TuJ1^+^ cells/location for each larvae at 3 dpf. One-way ANOVA with Dunn’s multiple comparisons test. N = 5. Error bars indicate standard error of the mean.

The overall morphology of transplanted AHPCs appeared similar to their original progenitor state *in vitro* at all three time points, with round soma and few short projections (Figs 3A’ and 4A”). Some cells exhibited neuronal phenotypes with a single long projecting process (Figs 3B’, 4A’ and 4B’). Immunohistochemical characterization was performed using markers for neural progenitors, neurons, astrocytes, and oligodendrocytes. Nestin immunolabeling for neural progenitors was commonly observed at all locations and time points (Fig 3). Quantification performed at 3 dpf indicated that approximately 50% of cells at each location were nestin-positive, with no significant difference among CNS, superficial, or other regions (N = 5) (Fig 4C, S5 Table).

Immunolabeling for the early neuronal marker TuJ1 detected differentiation of transplanted cells as early as 3 dpf (Fig 4). The CNS contained the highest percentage of TuJ1-expressing cells at 64% (Mean = 88.8, SD = 20, N = 6) (Fig 4.C, S6 Table). However, 75% of transplanted cells located in superficial regions were also positive for TuJ1 (Mean = 75, SD = 43.3, N = 5). Few cells in the other locations were immunolabeled for TuJ1 (Mean = 25, SD = 25, N = 3). No cells were positively labeled for the astrocyte marker GFAP or oligodendrocyte marker RIP at any time point.

**Fig 5:**
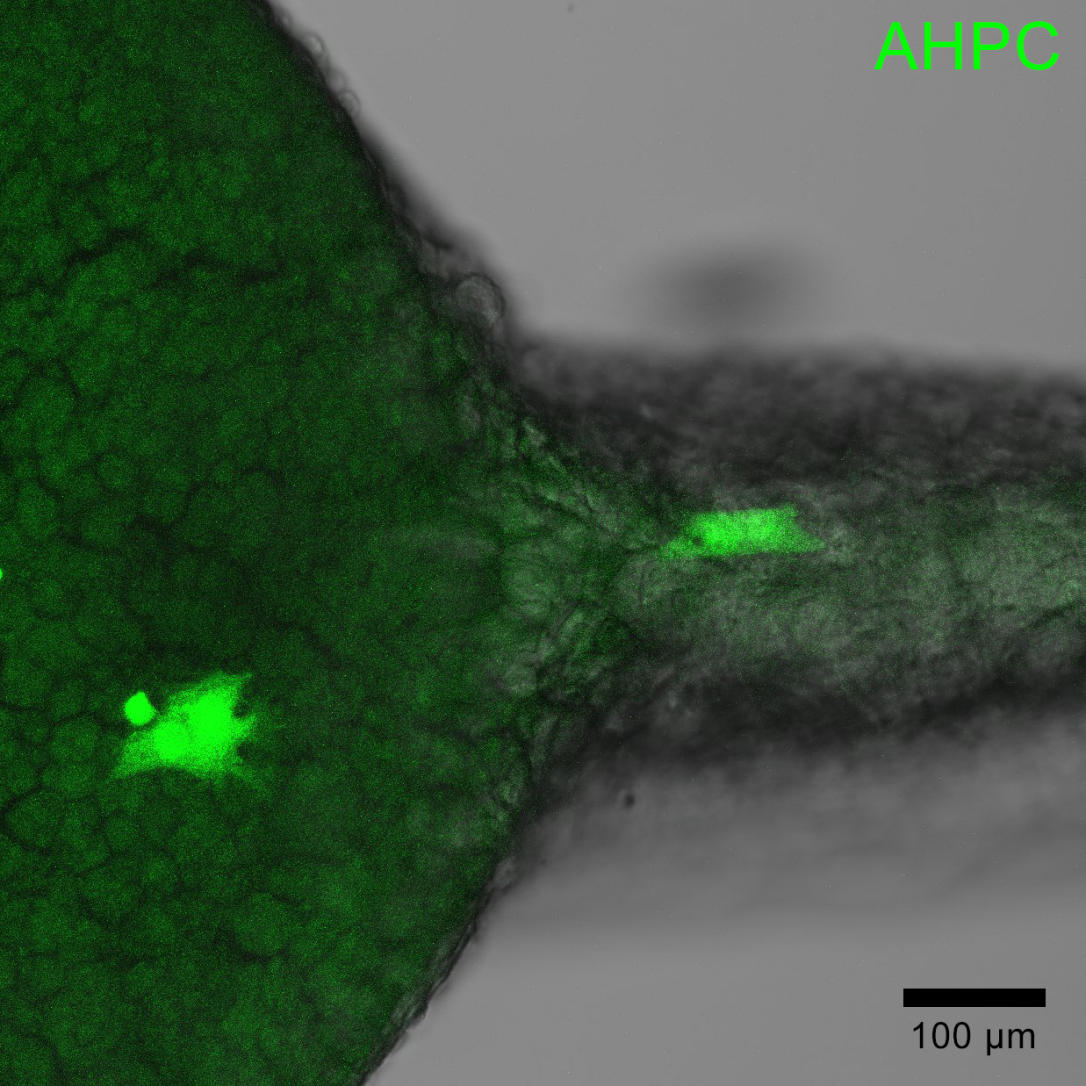
Representative image of transplanted AHPCs in the yolk periderm of a 1 dpf embryo exhibiting non-neural, flattened morphology.

A very small subset of superficially-located transplanted cells demonstrated unique morphology with flattened soma and lack of projections (Fig 5). However, this was only observed in 10 among 435 total cells.

## Discussion

In this study, adult rat hippocampal neural progenitors were transplanted into embryonic zebrafish to assess plasticity and potential impact of extrinsic versus intrinsic factors on cell fate. Xenografted cells were observed at least up to 5 days post-transplantation. Analysis of over 400 cells among 30 fish indicated that the relative proportion of AHPCs located in the CNS was significantly higher than those in other non-nervous regions by 5 dpf. A large proportion of transplanted cells were located at superficial regions such as epidermis and yolk periderm at all time points observed. However, AHPCs at superficial locations continued to display neural progenitor morphologies including round somata and one to two extended processes and positive immunolabeling for the neuronal marker TuJ1. Transplanted cells found at other non-nervous regions demonstrated similar neural characteristics. This extensive analysis utilizing immunohistochemistry of over 170 cells suggests that the transplanted progenitor cells did not morphologically incorporate into the animal or acquire alternative cell fates, with the exception of a very small percentage of cells acquiring unique flattened morphology.

This is the first case in which adult mammalian neural progenitor plasticity has been investigated by transplantation into embryonic zebrafish. Embryonic mouse neural progenitors have been transplanted into zebrafish at various stages in development by Xiao and colleagues [12]. When transplanted into 4 hpf blastulas, most cells were found in the CNS. Cells were also observed in mesoderm- and endoderm-derived tissues, but whether these cells acquired alternative fates was not determined. In contrast, immunohistochemistry performed in the present study determined that cell location did not appear associated with new fate. Even though a relatively equal proportion of cells were found outside versus within the CNS, a significant percentage of these cells in non-nervous regions were immunopositive for neural progenitor or neuronal markers.

After transplantation of embryonic neural progenitors by Xiao *et al*, some cells were found in the skin with epithelial morphology, though the percent of cells observed with this phenotype was not stated. When neural progenitors were co-cultured with mouse skin cells, they also began to express the epidermal marker keratin-1 [12]. The results of this paper report a very small percentage of adult neural progenitor cells exhibiting unique flattened morphology after transplantation. It may be that multipotent neural progenitors are capable of acquiring alternative fates, but at very low rates. Differences in observed cell fate may also be due to variance in plasticity between the embryonic neural progenitors used by Xiao and colleagues and the adult-derived neural progenitors used in this paper.

The ability for neural progenitor cells to demonstrate plasticity has varied depending upon origin of cells and the environment into which they were placed. Investigations of adult neural progenitor plasticity have utilized embryonic and adult progenitors, and transplantations have been performed within and across species. The origin of adult neural progenitors have also varied, including ependymal [3] and subventricular zones of the brain [13]. The impact of these differences on stem cell plasticity, as well as the possibility of cell fusion, are yet unknown. In addition to transplantation into embryonic animals, adult neural progenitor plasticity has also been observed after placement into adult tissues *in vivo*, such as bone marrow and skeletal muscle. Finally, co-culture of adult neural progenitor cells with various differentiated cells has resulted in skeletal, endothelial, epithelial, and myogenic differentiation, among others [14–17]. It is likely that variability in neural plasticity research is due to the origin of progenitor cells, such as differing neurogenic regions, and whether the cells are embryonic or adult-derived [18].

Approximately half of the transplanted adult neural progenitors described here retained expression of the neural progenitor marker nestin, indicating that many cells had not differentiated. Numerous findings of adult and embryonic neural progenitor plasticity *in vivo* and *in vivo* make it unlikely that the AHPCs transplanted in this study exhibited limited differentiation potential due to intrinsic genetic regulation. This lack of plasticity could be an effect of transplantation into zebrafish compared to chick or mouse embryos. Due to the very rapid development of zebrafish, transplanted cells may not have had sufficient opportunity to alter their fate *in vivo*. In adult mice, hippocampal neural progenitors begin expressing neuronal markers and morphology after two weeks [19]. *In vitro*, studies of rat adult hippocampal progenitors demonstrate neuronal fate six days after differentiation is induced [20]. However, zebrafish gastrulation and fate determination begins at 5 hpf, only two hours following transplantation of neural progenitors [9]. In a similar plasticity study, adipose-derived stem cells xenotransplanted into zebrafish blastulas were not yet differentiated at 2 dpf [21]. Transplantation of embryonic stem cells may help determine whether the developing zebrafish allows sufficient time for mammalian stem cell differentiation.

It is noted that the percent of transplanted AHPCs found in the CNS which were positive for the neuronal differentiation marker TuJ1 is in agreement with observations of AHPCs co-cultured with glial cells, a system which mimics the brain environment. *In vitro* differentiation procedures for AHPCs result in neurons, astrocytes, and oligodendrocytes within six days [20]. It appears that, at least for cells within the CNS, a short period of exposure to developmental factors in the zebrafish may be sufficient for differentiation.

The large number of cells observed in superficial regions of the zebrafish along with a general decrease in the total number of cells at each location following transplantation could also suggest that the cells were being expelled and/or dying. This loss of cells was most dramatic for cells in the superficial and other category, suggesting preferential survival of cells in the CNS. Similar results have been observed by Xiao and colleagues. When fetal mouse neural progenitors were transplanted into zebrafish, only ten percent of cells survived up to seven days [12]. It is unlikely that the cells were being rejected due to an immune response, as the zebrafish immune system is still immature throughout the time points observed [22]. Mammalian cancer cells have been transplanted into 2 dpf zebrafish at the yolk or orthotopic sites for observation of cancer cell invasion, or metastasis, and angiogenesis [23]. Further, neural progenitors lack immunogenicity, with no detectable expression of major histocompatibility complex class I or II [24, 25]. Alternatively, the neural progenitor cells may have been excluded from non-neural regions due to incompatibility with zebrafish cell adhesion molecules. Future experiments utilizing pluripotent embryonic stem cells may determine whether neural progenitors have developed characteristics which preclude them from full integration into the zebrafish.

The developing zebrafish may be an advantageous model for exploring plasticity of multipotent adult stem cells. Due to the zebrafish’s external development, transparent body, and immature immune system, transplanted progenitors can be easily tracked over a short period for assessment of cell fate. Adult rat neural progenitors survive transplantation into blastula-stage zebrafish and are observed at least five days throughout the organism. Besides the central nervous system, the majority of transplanted cells were located in superficial tissues of the zebrafish, such as epithelium. However, cell morphology and immunohistochemical analysis three days later indicate that approximately one-third of cells at this location retained neural fates rather than forming chimeric tissue. An extremely small proportion of transplanted cells located at the yolk periderm were observed with unique flattened, non-neural morphology, suggesting that adult neural progenitors may demonstrate some plasticity at a low rate in this system. The zebrafish model has potential for distinguishing enhanced differentiation potential for a variety of adult stem cells, helping to determine the role of extrinsic and intrinsic factors on cell fate.

## Supporting information

S1 Fig Zebrafish survive and developed normally at the elevated temperature of 34°C. A) Percent survival of animals at control (29°C) and elevated (34°C) temperature over time. N(control) = 57, N(treatment) = 71. B) Zebrafish development as measured by average animal length in mm. Note that initial reduced length at elevated temperature was recovered by 3 days post fertilization (dpf). p****< 0.0001. Two-way ANOVA with Tukey’s multiple comparisons test. N(control) = 7–19, N(treatment) = 16–22. Error bars indicate standard error of the mean.
(TIF)

S1 Table Quantification of zebrafish survival at 29 and 34°C. (XLSX)

S2 Table Quantification of zebrafish body length at 29 and 34°C. (XLSX)

S2 Fig Full-length images of zebrafish containing adult hippocampal progenitor cells up to 5 days post-transplantation. Green = GFP-expressing AHPCs. Scale bar = 500 μm.
(TIF)

S3 Fig The majority of AHPCs are nestin-positive *in vitro*. (TIF)

S3 Table Quantification of nestin-expressing AHPCs *in vitro*. (XLSX)

S4 Table Total number of cells located in the brain, superficial, or other regions per fish at 1, 3 and 5 days post-transplantation. (XLSX)

S5 Table Number of Nestin-positive cells per fish in the CNS, superficial, or other regions. (XLSX)

S6 Table Number of TuJ1-positive cells per fish in the CNS, superficial, or other regions. (XLSX)
