# Supplementary figures and images for: Xenotransplantation of adult hippocampal neural progenitors into the developing zebrafish for assessment of stem cell plasticity
Source: PLoS One. 2018 May 24;13(5):e0198025. doi: 10.1371/journal.pone.0198025 (PMC5967829; doi:10.1371/journal.pone.0198025)

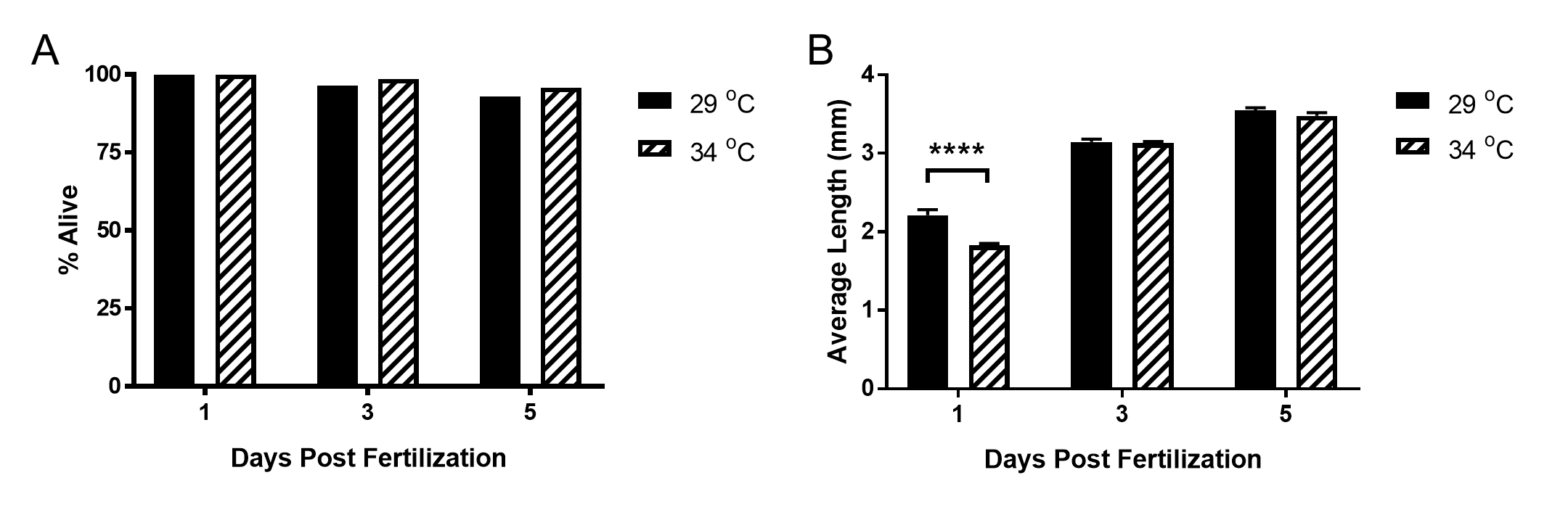

Supplement: S1 Fig — A) Percent survival of animals at control (29°C) and elevated (34°C) temperature over time. N(control) = 57, N(treatment) = 71. B) Zebrafish development as measured by average animal length in mm. Note that initial reduced length at elevated temperature was recovered by 3 days post fertilization (dpf). p****< 0.0001. Two-way ANOVA with Tukey’s multiple comparisons test. N(control) = 7–19, N(treatment) = 16–22. Error bars indicate standard error of the mean. (TIF) [file pone.0198025.s001.tif]

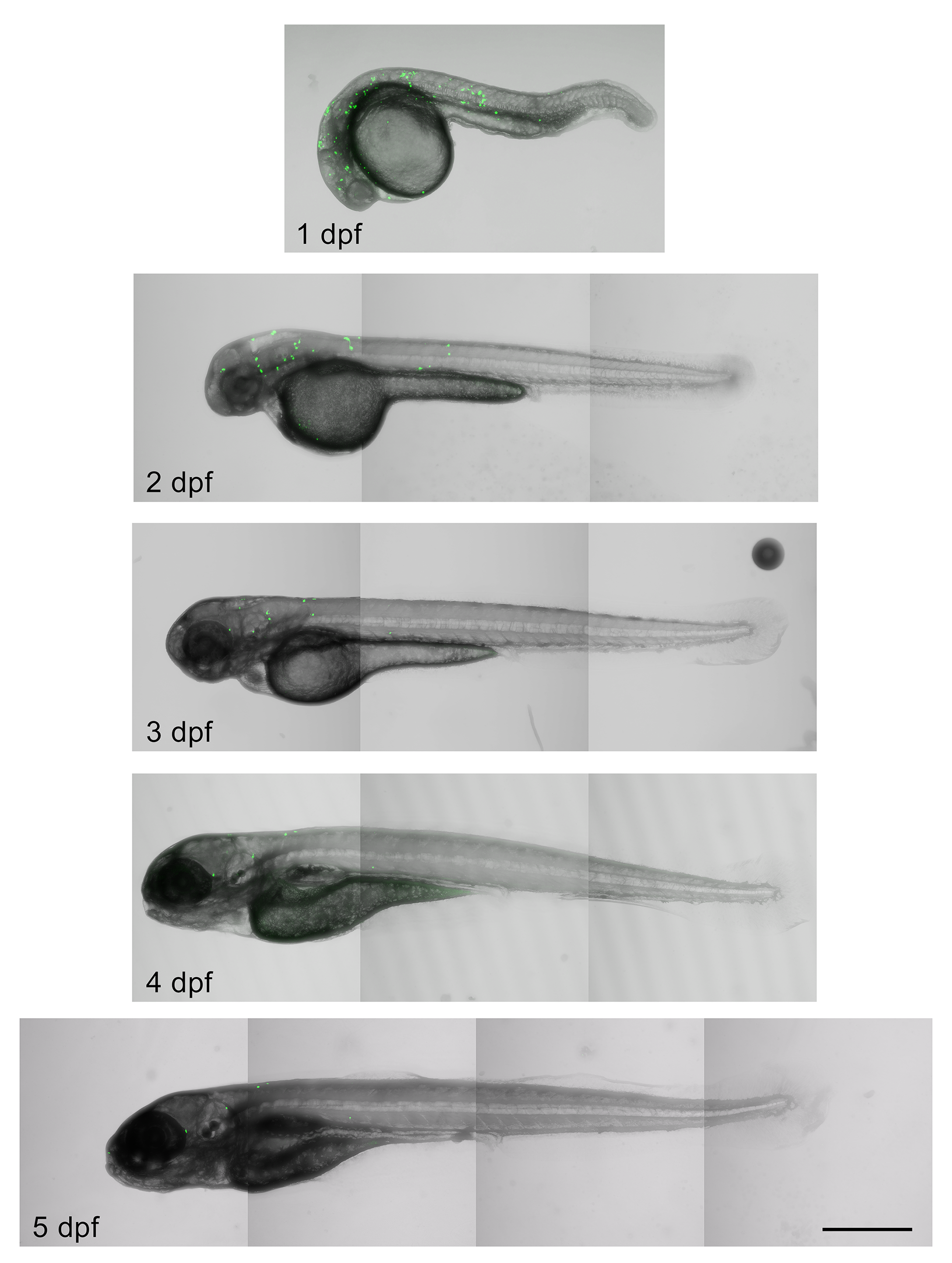

Supplement: S2 Fig — Green = GFP-expressing AHPCs. Scale bar = 500 μm. (TIF) [file pone.0198025.s004.tif]

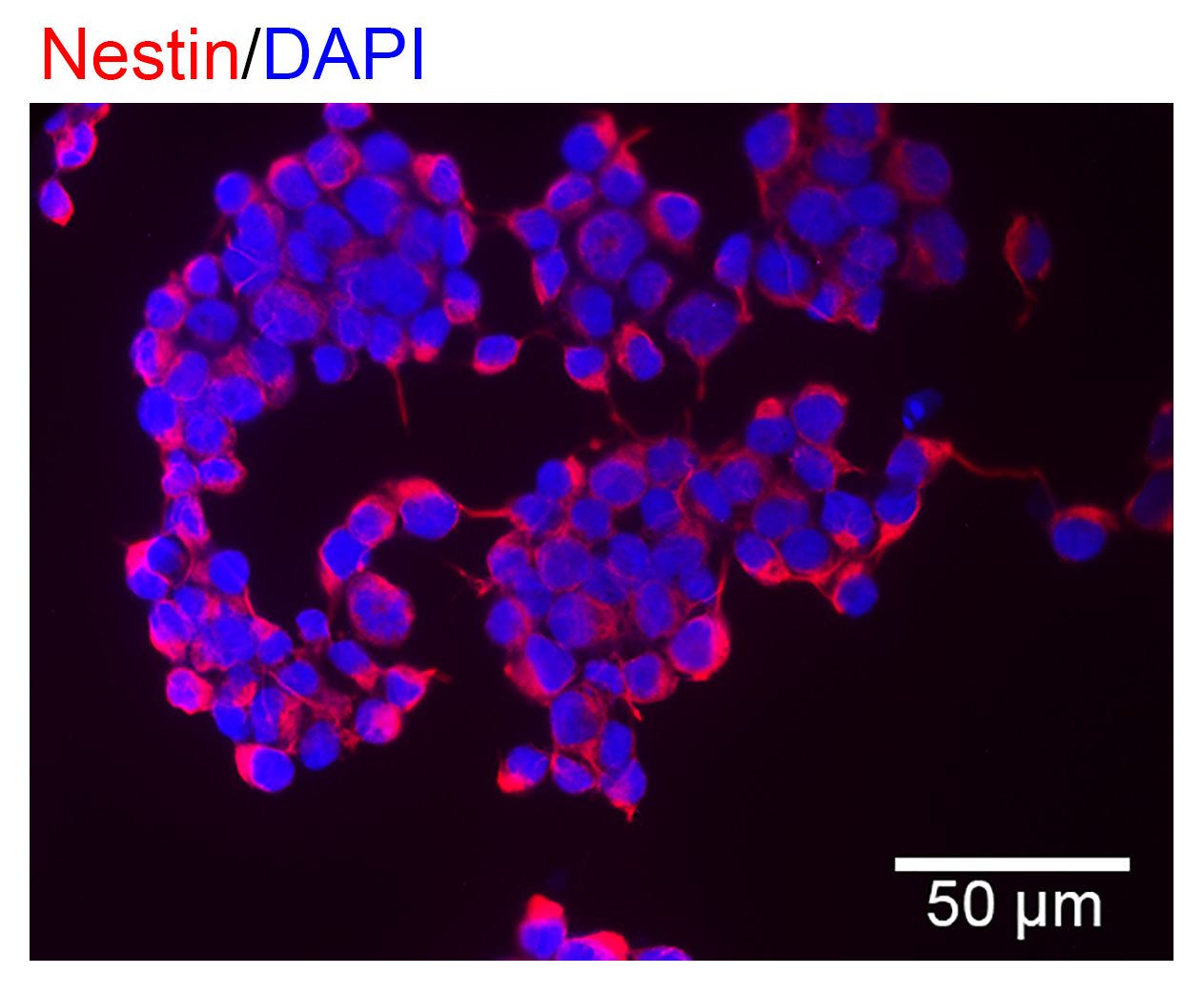

Supplement: S3 Fig — (TIF) [file pone.0198025.s005.tif]
